# Down-regulation of DANCR acts as a potential biomarker for papillary thyroid cancer diagnosis

**DOI:** 10.1042/BSR20181616

**Published:** 2019-04-17

**Authors:** Ke Zhang, Jing Lv, Xiaowei Peng, Jianqiu Liu, Cuilin Li, Jing Li, Ningwei Yin, Hui Li, Zhi Li

**Affiliations:** 1 Department of Clinical Pharmacology, Xiangya Hospital, Central South University, 87 Xinagya Road, Changsha 410008, P.R. China; 2 Institute of Clinical Pharmacology, Central South University, Hunan Key Laboratory of Pharmacogenetics,110 Xiangya Road, Changsha 410078, P.R. China; 3 Engineering Research Central of Applied Technology of Pharmacogenomics, Ministry of Education, 110 Xiangya Road, Changsha 410078, P.R. China; 4 National Clinical Research Center for Geriatric Disorders, 87 Xiangya Road, Changsha 410008, Hunan, P.R. China; 5 Department of Endocrinology, Zhengzhou Central Hospital, Zhengzhou University, Zhengzhou 450007, P.R. China; 6 Department of Surgery, Zhengzhou Central Hospital, Zhengzhou University, Zhengzhou 450007, P.R. China

**Keywords:** DANCR, diagnosis, large intervening non-coding RNA, thyroid cancer

## Abstract

Long non-coding RNAs (lncRNAs) have been reported to be dysregulated and play a crucial role in the progression of cancer. LncRNA DANCR has recently been revealed to be involved in tumorigenesis of numerous types of cancer, including osteosarcoma, gastric cancer, breast cancer, hepatocellular carcinoma, and colorectal cancer. However, the expression profiles and biological relevance of DANCR in papillary thyroid cancer (PTC) have not yet been reported. In the present study, the expression level of DANCR in PTC tissues and adjacent normal tissues was detected by reverse transcription-quantitative PCR in PTC patients, and then we analyzed the association with clinical pathological characteristics of patients and DANCR expressions. These results demonstrated that the expression of DANCR was notably decreased in tumor tissues in comparison with adjacent normal tissues (*P*<0.001). Furthermore, the present study found that DANCR expression level was correlated to T grade (*P*<0.01) and TNM stage (*P*=0.017). The present study demonstrated that DANCR was associated with PTC aggressive clinical features and may serve as a diagnostic biomarker for detecting PTC patients.

## Introduction

Although the incidence of thyroid cancer is rapidly increased, the mortality rate remains flat [1,2]. But the current debate is how to perform clinical treatment, because traditional treatment is controversial and involves over-diagnosis [3,4]. (Although the incidence of thyroid cancer is rapidly increased, the mortality rate keeps flat [1,2]. Some stuides suggested that trends in PTC incidence may be explained by two underlying processes: the dominat one is overdiagnosis, and the other one is a small but actual increase in PTC incidence.) As shown above, despite advances made in diagnosis for papillary thyroid cancer (PTC), the incidence of PTC remain high [5]. Although numerous researches elucidated that BRAF mutation is associated with invasive behavior of PTC and proposed that BRAF mutation would be a prognostic biomarker, there is a controversy because the positive ratio of BRAF mutation in PTC can reach more than 50%, while – in fact – only about 10–15% of PTC patients show aggressive biological behaviors [6–8].Therefore, it is necessary to find novel biomarkers to improve diagnosis accuracy between high-aggressive and low-aggressive PTC patients.

Long non-coding RNAs (lncRNAs) constitute a newly identified class of RNAs, with more than 200 nucleotides in length and regulating gene expression through controlling transcription or post-transcription, epigenetic modification, and mRNA splicing [9,10]. Although not translated to proteins, lncRNAs have critical roles in cellular and physiologic functions, for instance, cell proliferation, differentiation, and apoptosis [11]. Particularly, lncRNAs are crucial player in regulating cancer-related gene expression [12]. Recent articles demonstrated that dysregulation of lncRNAs contributes to cancer progression through endogenous-sponging miRNAs or direct-binding miRNAs. In addition, lncRNAs could act as diagnostic or prognostic biomarkers in various cancers [13–17]. LncRNA, anti-differentiation non-coding RNA (lncRNA DANCR or ANCR) was required for de-differentiation of epidermal cells [18]. Ma et al. [19] found that DANCR promotes tumor growth and metastasis and acts as a diagnostic biomarker in hepatocellular carcinoma. Jiang et al. [20] demonstrated that DANCR promotes osteosarcoma progression by up-regulating AXL via inhibiting the expression of miR-33a-5p. Recently, Ma et al. [21] found that DANCR promotes cisplatin resistance via activating AXL/PI3K/Akt/NF-κB signaling pathway in glioma. However, the role of DACNR in thyroid cancer is poorly understood.

By analyzing the GEO datasets, we found DANCR is significantly decreased in PTC tissues compared with normal adjacent tissues. In the present study, we detected the expression of DANCR in PTC tissues and adjacent normal tissues, and then investigated the correlation between DANCR expression and clinicopathologic characteristics. Finally, we investigated the feasibility of using DANCR to detect PTC patients.

## Methods

### Expression analysis in GEO database

Three expression profiles (GSE33630 [22], GSE50901 [23], and GSE66783 [24]) were downloaded from Gene Expression Omnibus (GEO; http://www.ncbi.nlm.nih.gov/geo/). GSE33630 was based on Affymetrix Human Genome U133 Plus 2.0 Array (Affymetrix Inc., Santa Clara, CA, U.S.A.), GSE50901 was based on Agilent-028004 SurePrint G3 Human GE 8×60K Microarray (Agilent Technologies, Santa Clara, CA, U.S.A.), and GSE66783 was based on Agilent-060228 Human LncRNA v4 4×180K (Agilent Technologies, Santa Clara, CA, U.S.A.). In GSE33630, 49 PTC and 45 normal thyroid tissues were analyzed. A total of 57 PTC and nine adjacent normal thyroid tissues were employed in GSE50901; five PTC specimens and their paired adjacent noncancerous thyroid tissue samples were obtained in GSE66783. In order to obtain the expression of DANCR in GEO datasets, GEO2R was used to analyze the DANCR expression in PTC and normal tissues and the profile graph was employed to visualize DANCR expression in each sample.

### Patients and tissue samples

Altogether, 96 fresh thyroid cancer tissues and paired adjacent normal tissues were obtained from patients who had undergone surgical resection of thyroid cancer from July 2016 to June 2017 at the Hunan Cancer Hospital of Central South University, China. Thyroid cancer were diagnosed and graded by the Pathology Department of Hunan Cancer Hospital according to the TNM stage standard. With medical history in consideration, only 76 patients have been enrolled into this study. No patients had been treated with radiotherapy or chemotherapy before surgery. This study was approved by the Ethics Committee of Hunan Cancer Hospital of Central South University and informed consent form (IFC) was signed by all patients involved in the study. Tissue samples were stored in liquid nitrogen immediately after surgery until total RNA was extracted. Clinical characteristics, such as age, gender, and TNM stage, were all collected from the case management system in Hunan Cancer Hospital.

### RNA extraction and quantitative real-time PCR (qRT-PCR)

Total RNA was extracted from tissue specimens by TRIzol reagent (Invitrogen) according to the Invitrogen manufacturer’s protocol as done in previous research [25]. The quality and quantity of extracted RNA was measured by a NanoDrop Spectrophotometer (Shimadzu Biotech, Beijing, China).The extracted RNA was determined to be pure only when the A260/A280 ratio is 1.8 to 2.1. The isolated RNA concentration was calculated and normalized with RNase-free water and then reverse-transcribed into cDNA using PrimeScript™ RT reagent kit with gDNA Eraser (RR047A; Takara, Dalian, China). All cDNA samples were stored at -80°C until use. The quantitative real-time PCR (qRT-PCR) was conducted by Light Cycle® 480 II (Roche, Basel, Switzerland) by using a SYBR® Premix DimerEraser™ (Perfect Real Time) (Code No: RR091A, Takara Bio Inc.) in a 20 µl mixture according to the manufacturer’s protocol. The qRT-PCR amplification procedure was performed as follows: an initial denaturation at 95°C for 30 s, followed by 40 cycles at 95°C for 5 s, 60°C for 30 s, and 72°C for 30 s. The relative expression of target gene was normalized to the endogenous gene β-actin. We used the following equation to calculate the relative expression of DANCR: 2^−ΔΔC^_t_ = ([C_t_ DANCR-C_t_ β actin] sample A − [C_t_ DANCR-C_t_ β actin] sample B) (sample A is the cancer tissue, sample B is the adjacent normal tissue) [26]. All reactions were operated repetitively. The primer sequencing of lncRNA DANCR and β-actin were synthesized by Sangon Bio-tech (Shanghai, China).

The primer sequencing results were as follows:

DANCR sense: 5′- TCGGAGGTGGATTCTGTT -3′;

DANCR anti-sense: 5′- TTCGGTGTAGCAAGTCTG -3′;

β-actin sense: 5′- CCTGGCACCCAGCACAAT -3′;

β-actin anti-sense: 5′-GGGCCGGACTCGTCATAC -3′.

### Statistical analysis

We used SPSS version 19.0 software (IBM Corp, Chicago, IL, U.S.A.) to analyze data and used GraphPad Prism V.7.00 software (GraphPad Software, La Jolla, CA, U.S.A.) to draw figures. Paired Student’s *t* test was used to compare the difference in DANCR expression between tumor tissues and paired adjacent normal tissues. Non-parametric Mann–Whitney *U* test was used to compare the differences in DANCR expression between the tumor and healthy groups in GEO datasets. The DANCR expression levels in PTC tissues were categorized as low expression and high expression, in relation to mean value. Chi-square (χ^2^) test was applied to determine the association between the DANCR expression and the clinicopathological features of PTC. Multivariable logistic regression analysis was used to evaluate whether DANCR is an independent factor for TNM staging or not and data were expressed with odds ratio (OR) and 95% confidence interval (95% CI). Receiver operating characteristic (ROC) curve and area under the ROC curve (AUC) were used to evaluate the feasibility of using DANCR to detect PTC. *P*-value <0.05 was considered as a statistically significant difference.

## Results

### DANCR expression was decreased in tissue from PTC patients

**Figure 1:**
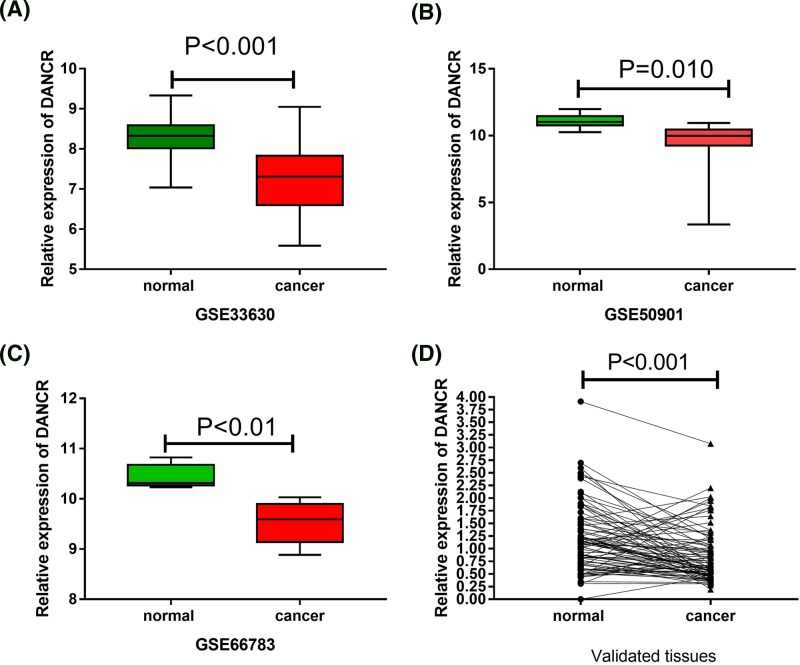
The expression of DANCR in GEO dataset and clinical data (**A**) GSE33630, (**B**) GSE50901, (**C**) GSE66783, and (**D**) clinical data. Notes: Data were analyzed by Paired Student’s *t* test and non-parametric Mann–Whitney *U* test. ^*^P<0.05, ^**^P<0.01, and ^***^P<0.001.

Before the experiments, we compared the expression of DANCR between PTC and non-tumor thyroid tissues in three GEO datasets (GSE33630, GSE50901, and GSE66783). As shown in Figure 1A–C, DANCR expression was significantly decreased in PTC tissues compared with non-tumoral thyroid tissues in these datasets (*P*<0.001). To further confirm the expression of DANCR in PTC tissues and matched adjacent normal tissues, qRT-PCR was used to detect the expression of DANCR in 76 paired PTC tissues and adjacent non-cancerous tissues. As shown in Figure 1D, the expression of DANCR was significantly decreased in PTC (*P*<0.001) compared with normal counterparts, which was in accordance with the analysis in GEO datasets.

### Correlations between lncRNA DANCR expression and clinicopathologic features in PTC patients

**Figure 2:**
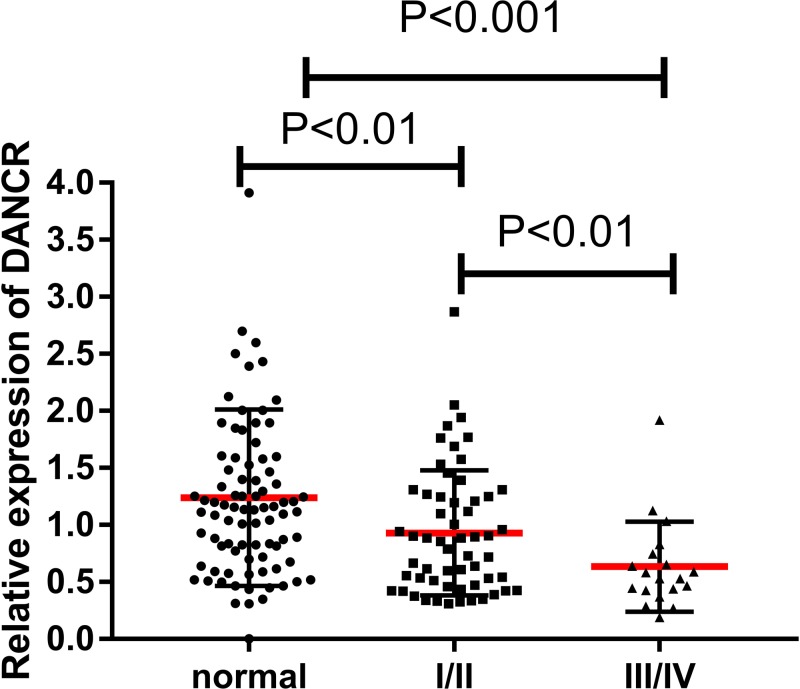
The expression of DANCR in normal tissues and different TNM stages of PTC patients Notes: Normal controls (*n*=76) and all thyroid cancers including I/II (*n*=57), III/IV (*n*=19). Data were analyzed by Paired Student’s *t* test and non-parametric Mann–Whitney *U* test.

To analyze whether DANCR expression was associated with the progression of PTC or not, we investigated the relationship between DANCR expression and clinical features. Patients were divided into two groups (high DANCR expression group [*n*=38] and low DANCR expression group [*n*=38]) based on the median value (0.668).The relationship between DANCR and clinical characteristics of PTC was analyzed by χ^2^ test and summarized in Table 1. After χ^2^ test, we found that the expression of DANCR was significantly associated with TNM stage (*P*<0.05) and T stage (*P*<0.01). However, there was no significant correlation of DANCR expression with other clinical features such as gender, age, tumor size, and local invasion (*P*>0.05). DANCR expression was significantly decreased in III/IV stage thyroid cancer compared with I/II stage thyroid cancer and normal tissues in 76-pair samples (Figure 2). In other words, DANCR expression was negatively correlated with clinical stage/aggressive degree of thyroid cancers. As shown in Table 2, multivariable analysis showed that DANCR is an independent protective factor for TNM stage (β<0, OR<1) excluding other factors, such as age, sex, BMI, and serum TSH levels.

**Table 1:** The relationship between DANCR expression and clinicalpathological factors in thyroid cancer

Characteristics	Number	lncRNA DANCR		χ^2^	*P*-value
		Low (%)	High (%)		
Age (years)					
<45	44	26 (59.10)	18 (40.90)	3.455	0.063
≥45	32	12 (36.36)	20 (63.64)		
Gender					
Male	15	7 (46.67)	8 (53.33)	1.016	0.602
Female	61	31 (50.82)	30 (49.18)		
Tumor size (cm)					
<2	44	26 (59.09)	18 (40.91)	3.455	0.063
≥2	32	12 (37.5)	20 (62.5)		
T stage					
T1–T2	61	25 (40.98)	36 (59.01)	10.05	0.002**
T3–T4	15	13 (86.67)	2 (13.33)		
TNM stage					
I/II	57	24 (42.11)	33 (57.89)	5.684	0.017*
III/IV	19	14 (73.68)	5 (23.62)		
Extrathyroidal extension					
Yes	40	21 (52.50)	19 (47.50)	1.317	0.251
No	36	17 (47.22)	19 (52.78)		
Lymph node metastasis					
N0	41	20 (48.78)	21 (51.22)	0.071	0.790
N1	35	16 (45.71)	19 (54.29)		

Notes: Data were analyzed by chi-square (χ^2^) test and by Student’s *t* test, given as mean ± SD.

^*^P<0.05, ^**^P<0.01.

**Table 2:** Association between TNM stage and lncRNA DANCR expression by multivariate analysis

	LncRNA DANCR		
	OR	95% CI	*P*-value
PTC diagnosis			
Model 1	0.26	0.082–0.819	0.021*
TNM stage			
Model 1	0.028	0.003–0.266	0.002**
Model 2	0.015	0.001–0.229	0.003**

Notes: Model 1, adjusted for sex and age. Model 2, adjusted for sex, age, tumor size, lymph node metastasis, and thyroid stimulating hormone pre-operation.

^*^P<0.05, ^**^P<0.01.

### The diagnosis value of DANCR in PTC

**Figure 3:**
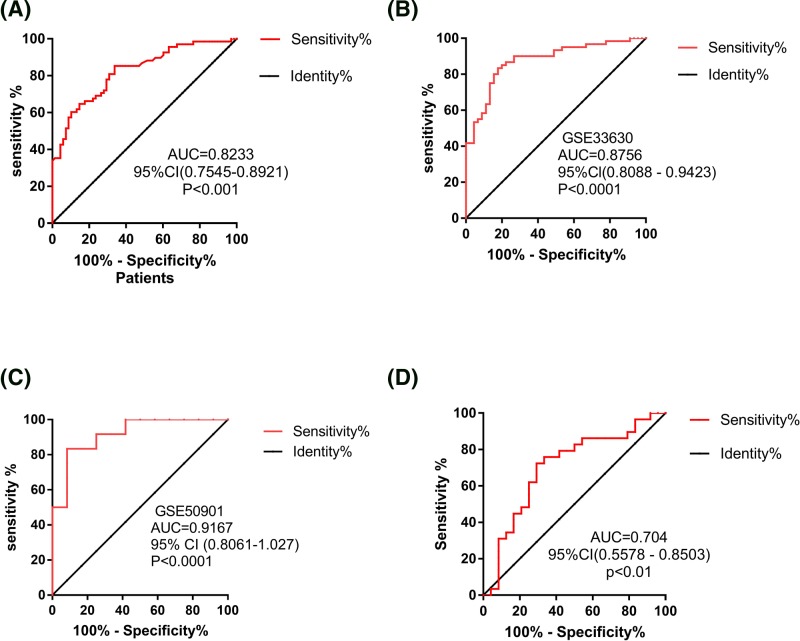
ROC of tissue DANCR expression for differentiating PTC tissue from normal tissue and for differentiating early-stage from advanced-stage patients Notes: The ROC analysis of DANCR for PTC patients in 76-pair tissues (**A**), GSE33630 (**B**), and GSE50901 (**C**). The ROC analysis for detection of PTC patients with TNM stage III–IV from those with TNM stage I–II using DANCR (**D**).

ROC curve and AUC were used to investigate characteristics of the DANCR as potential tumor markers of PTC on data from all subjects, including 76 PTC patients’ tissues and 76 adjacent normal thyroid tissues, PTC and normal thyroid tissues in GSE33630 and GSE50901. The ROC curves illustrated strong separation between the PTC patients and adjacent normal group, with an AUC of 0.8233 (sensitivity was 85.29% and specificity was 66.18%; Figure 3A) for DANCR in 76-pair samples. The values of AUC of thyroid cancer versus healthy controls in GSE33630 and GSE50901 were 0.8756 (sensitivity was 81.54% and specificity was 82.22%; Figure 3B) and 0.9167 (sensitivity was 83.33% and specificity was 91.67%; Figure 3C), respectively. Furthermore, in order to explore whether there is a correlation between DANCR expression and TNM staging, we also evaluated the expression of DANCR in different TNM staging of PTC patients. These results showed that DANCR was significantly down-regulated in patients with III/IV staging compared with patients with I/II staging (*P*<0.05, Figure 2). Moreover, DANCR could discriminate PTC I/II patients from III/IV patients with AUC 0.704 (sensitivity was 72.41% and specificity was 70.83%; Figure 3D). Therefore, DANCR provided the promising diagnostic power for the detection of PTC, suggesting that DANCR could serve as a potential tumor marker for PTC diagnosis.

## Discussion

Recently, many studies have demonstrated the functions of lncRNAs in different types of cancer and evaluated their potential implications in diagnosis and therapy [12]. Although numerous studies have focused on lncRNAs as potential tumor markers for cancer diagnosis and prognosis prediction, the diagnostic utility of DANCR in PTC has never been studied.

In the present research, we identified that lncRNA DANCR expression was significantly decreased in 76 PTC patients’ tissues compared with adjacent normal tissues. Moreover, we found that the expression of DANCR was negatively correlated with T staging and TNM staging in 76 PTC patients. Furthermore, multivariable logistic regression analysis indicated that DANCR was an independent protective factor for TNM staging (β<0, OR<1). Finally, DANCR could discriminate PTC from normal adjacent tissues with AUC of 0.8233 (95% CI: 0.7545–0.8921, *P*<0.0001), these findings provided evidences for detecting PTC by using DANCR as a novel diagnostic marker.

Previous functional studies on lncRNA provide evidence that DANCR is a tumor-associated lncRNA and up-regulated in numerous human cancers, including breast cancer, prostate cancer, gastric cancer, and osteosarcoma [27]. DANCR is a lncRNA that acts as a negative regulator of cell differentiation. Jiang et al. [20] demonstrated that DANCR increases CSC function by up-regulating AXL via competitively binding to miR-33a-5p, and this function is sequentially performed through the PI3K-Akt signaling pathway. In another similar study, Lu et al. [27] demonstrated that DANCR promotes lung adenocarcinoma progression via modulating the expression levels of mTOR by directly binding to miR-496. Although many studies have demonstrated that DANCR serves as a tumorigenic factor and could promote tumor invasion and metastasis in various cancers, Zhong et al. [28] found that DANCR worked as a tumor suppressor by controlling the EMT process and cancer cell migration. Similar to the Zhong et al. study, we found that DANCR is low expressed lncRNA in PTC tissues and negatively associates with aggressive clinical features. There are some potential mechanisms to explain the different expression of DANCR in thyroid cancer. Zhu et al. [29] demonstrated that DANCR associates with enhancer of zeste homolog 2 (EZH2) to repress expression of the runt-related transcription factor 2 gene (Runx2). Dalle et al. [30] found that Runx2 mRNA expression was higher (7.81-fold expression) in PTC than in normal tissue. Niu et al. [31] study suggested that enhanced Runx2 was functionally linked to tumor invasion and metastasis of thyroid carcinoma by regulating EMT-related molecules, matrix metalloproteinases, and angiogenic/lymph-angiogenic factors. Therefore, DANCR may perform different function and expression in different cancer types and cancer cell lines.

However, our study still contains several limitations. For one thing, the diagnostic efficiency of DANCR should be based on large clinical samples. Therefore, in future experiments, we need to expand the sample size and continue to verify its value. For another thing, *in vitro* and *in vivo* experiments needed to be conducted to further validate the biological function of DANCR in thyroid cancer. Understanding the underlying mechanism of DANCR in PTC will be helpful to establish the novel diagnostic biomarker.

## Conclusion

Overall, our results suggested that decreased DANCR can be used as a potent tumor biomarker for PTC detection, and DANCR is an independent factor for TNM stage.

## Abbreviations

Akt: protein kinase B
AUC: area under the ROC curve
AXL: Anexelekto
BRAF: V-raf murine sarcoma viral oncogene homolog B1
CI: confidence interval
CSC: cancer stem cell
EMT: epithelial–mesenchymal transition
gDNA: genomic DNA
lncRNA: long non-coding RNA
PI3K: phosphatidylinositol 3 kinase
PTC: papillary thyroid cancer
qRT-PCR: quantitative real-time PCR
ROC: receiver operating characteristic
Runx2: runt-related transcription factor 2 gene
TNM: tumor node metastasis
TSH: thyroid stimulating hormone
